# Clinical diagnosis of Larsen syndrome, Stickler syndrome and Loeys-Dietz syndrome in a 19-year old male: a case report

**DOI:** 10.1186/s12881-018-0671-0

**Published:** 2018-08-31

**Authors:** N. Riise, B. R. Lindberg, M. A. Kulseth, S. O. Fredwall, R. Lundby, M.-E. Estensen, L. Drolsum, E. Merckoll, K. Krohg-Sørensen, B. Paus

**Affiliations:** 1 TRS National Resource Centre for Rare Disorders, Sunnaas Rehabilitation Hospital, Nesoddtangen, N-1450 Oslo, Norway; 2 Department of Cardiothoracic Surgery, Oslo University Hospital, Oslo, Norway; 3 Department of Medical Genetics, Oslo University Hospital, Oslo, Norway; 4 Department of Radiology and Nuclear Medicine, Oslo University Hospital, Oslo, Norway; 5 Department of Cardiology, Oslo University Hospital, Oslo, Norway; 6 Department of Ophthalmology, Oslo University Hospital, Oslo, Norway; 7 Institute of Clinical Medicine, University of Oslo, Oslo, Norway

**Keywords:** Larsen syndrome, Stickler syndrome, Loeys-Dietz syndrome, High throughput sequencing

## Abstract

**Background:**

Larsen syndrome is a hereditary disorder characterized by osteochondrodysplasia, congenital large-joint dislocations, and craniofacial abnormalities. The autosomal dominant type is caused by mutations in the gene that encodes the connective tissue protein, filamin B (*FLNB*). Loeys-Dietz syndrome (LDS) is an autosomal dominant connective tissue disorder characterized by arterial aneurysms, dissections and tortuosity, and skeletal, including craniofacial, manifestations. Mutations in five genes involved in the transforming growth factor beta (TGF-β) signaling pathway cause five types of LDS. Stickler syndrome is a genetically heterogeneous arthro-ophthalmopathy caused by defects in collagen, exhibiting a wide specter of manifestations in connective tissue. A rare case is reported that was diagnosed with all these three hereditary connective tissue disorders.

**Case presentation:**

A 19 year-old, Norwegian male with a clinical diagnosis of Larsen syndrome and with healthy, non-consanguineous parents attended a reference center for rare connective tissue disorders. Findings at birth were hypotonia, joint hypermobility, hyperextended knees, adductovarus of the feet, cervical kyphosis, craniofacial abnormalities, and an umbilical hernia. From toddlerhood, he required a hearing aid due to combined conductive and sensorineural hearing loss. Eye examination revealed hyperopia, astigmatism, and exotropia. At 10 years of age, he underwent emergency surgery for rupture of an ascending aortic aneurysm. At 19 years of age, a diagnostic re-evaluation was prompted by the findings of more distal aortic dilation, tortuosity of precerebral arteries, and skeletal findings. High throughput sequencing of 34 genes for hereditary connective tissue disorders did not identify any mutation in *FLNB*, but did identify a de novo missense mutation in *TGFBR2* and a nonsense mutation in *COL2A1* that was also present in his unaffected father. The diagnosis was revised to LDS Type 2. The patient also fulfills the proposed criteria for Stickler syndrome with bifid uvula, hearing loss, and a known mutation in *COL2A1*.

**Conclusion:**

LDS should be considered in patients with a clinical diagnosis of Larsen syndrome, in particular in the presence of arterial aneurysms or tortuosity. Due to genetic heterogeneity and extensive overlap of clinical manifestations, genetic high throughput sequencing analysis is particularly useful for the differential diagnosis of hereditary connective tissue disorders.

## Background

Larsen syndrome was first described in 1950 as a syndromic form of osteochondrodysplasia comprising congenital dislocations of the hip, knee, and elbow joints; scoliosis; cervical kyphosis; equinovarus or equinovalgus foot deformities; spatula-shaped fingers; and characteristic craniofacial abnormalities. Useful diagnostic features in early childhood include supernumerary carpal and tarsal bones as well as an extra calcaneal ossification center appearing in late infancy, fusing with the main ossificiation center at about 8 years of age. Dysmorphic features include hypertelorism, prominent forehead, depressed nasal bridge, flattened midface, and cleft palate [1]. Hearing loss is a well-recognized complication of Larsen syndrome. Inheritance is usually autosomal dominant with pronounced variable expressivity. In 2007, Bicknell et al. identified heterozygosity for mutations in the gene *FLNB*, which encodes the protein filamin B, in 20 unrelated patients with Larsen syndrome [2]. Filamin B may affect cytoskeleton-dependent cell proliferation, differentiation, and migration,

Larsen syndrome belongs to the group of hereditary connective tissue disorders which comprises more than 40 distinct monogenic disorders. Many of them are autosomal dominantly inherited with highly variable expressivity and extensive overlapping of clinical manifestations [3, 4], especially involving the musculoskeletal, cardiovascular, respiratory, ophthalmologic, and cutaneous systems. Diagnostic accuracy is most critical for serious and treatable disorders [4]. Many hereditary connective tissue disorders are defined as genetic syndromes, which implies the use of clinical diagnostic criteria. In the past decade, the identification of a number of causative genes and the development of more efficient genetic sequencing analysis have also enabled molecular diagnosis in most of the disorders.

Loeys-Dietz syndrome is a connective tissue disorder characterized by cardiovascular manifestations (most often thoracic, abdominal, and cerebral arterial aneurysms and/or dissections) in addition to skeletal manifestations such as pectus excavatum or carinatum, scoliosis, joint laxity, arachnodactyly, talipes equinovarus, and craniofacial manifestations (hypertelorism, bifid uvula or cleft palate, and/or craniosynostosis). Following the identification of the syndrome in 2005 [5], five different subtypes of LDS as well as five different causative genes have been described [5–9].

## Case presentation

A Norwegian 19-year old male with healthy, non-consanguineous parents attended a reference center for rare connective tissue disorders with a diagnosis of Larsen syndrome. The diagnosis was based on clinical findings in the neonatal period. He was born with dislocated, hyperextended knees up to 90 degrees, which was treated with serial casting without success. This treatment was followed by surgery with Ilizarov’s frame and braces at 9 months of age. Adductovarus of the feet responded well to non-surgical treatment. His neck was kyphotic with a subluxation of C3-C4 and dislocations of C4-C5 and C5-C6. A fixation from C1 to C5 was performed bilaterally using a bone graft from his rib when he was 16 months old. A small mandible and occult cleft palate were observed. Cerebral ultrasound showed some dilatation of the ventricular system. Hypotonia and joint hypermobility of the knees, ankles, and wrists were also observed. In addition, he had an umbilical hernia and a large, left medial inguinal hernia.

As a toddler, he had recurrent serous otitis media and was examined by an ENT consultant. A combined conductive and sensorineural hearing loss was detected and necessitated a hearing aid. The patient was followed by an ophthalmologist because of hyperopia, astigmatism, and exotropia. His hyperopia was corrected with glasses, and the exotropia was treated with patching and strabismus surgery. Further, he has asthma and atopic eczema.

At 10 years of age, the patient was admitted to the hospital with acute chest pain. Echocardiography and CT showed a dilated and dissected aortic root to 7 cm, aortic valve regurgitation, and hemopericardium but normal dimensions in the aortic arch. Emergency surgery to replace the aortic root with a mechanical valve was performed. Following surgery, the patient was treated with beta-blockers and warfarin. At this point a PubMed search was carried out. According to his medical records, one not named case article about a child with Larsen syndrome and arterial tortuosity and dilatation was found. No clinical diagnostic screening was performed.

**Fig. 1:**
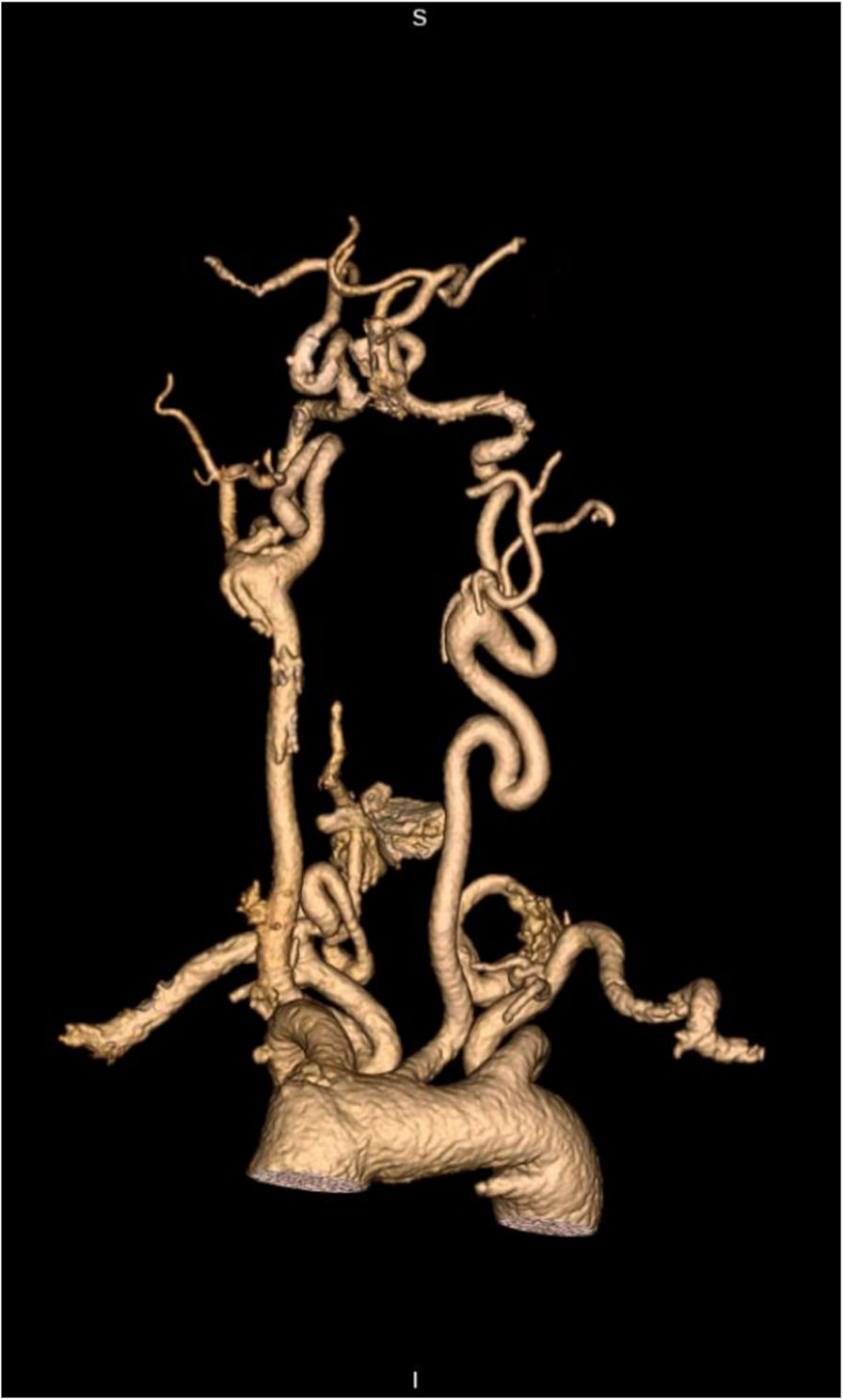
MRI angiogram of the patient’s vertebral, carotid and subclavian arteries at the age of 19, demonstrating severe arterial tortuosity. Permission to publish the image was given from the Department of Radiology and Nuclear Medicine at Oslo University Hospital

At age 19, MR angiography revealed a dilatation of 44 mm of the ascending aorta and arch distal to the graft. There was also severe tortuosity of the vertebral, carotid, and subclavian arteries (Fig. 1) and a moderate dilatation of the left iliac artery. According to guidelines [10], surgery is recommended at ascending aortic diameter of 42 mm in LDS. The patient has now undergone aortic arch surgery.

**Fig. 2:**
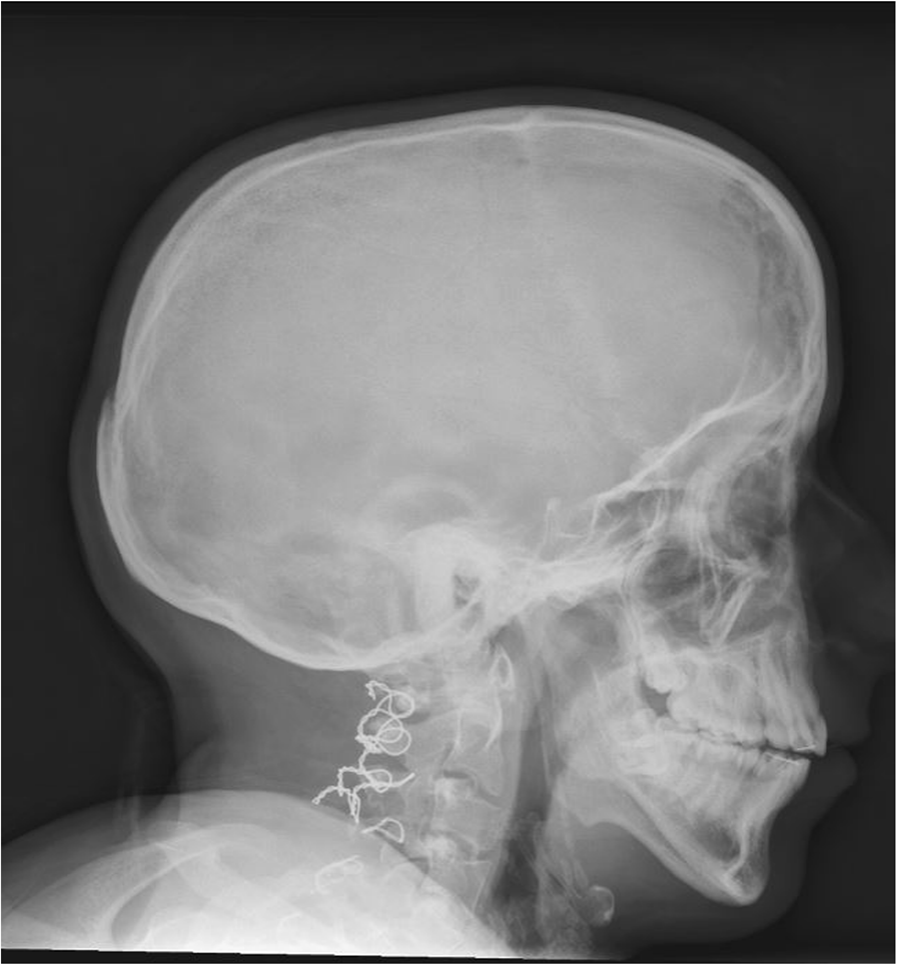
Lateral cranium and upper cervical spine at 20 years showing subtle micrognathia, cervical platyspondyly and metal sutures from previous surgery located posteriorly over the upper spine

**Fig. 3:**
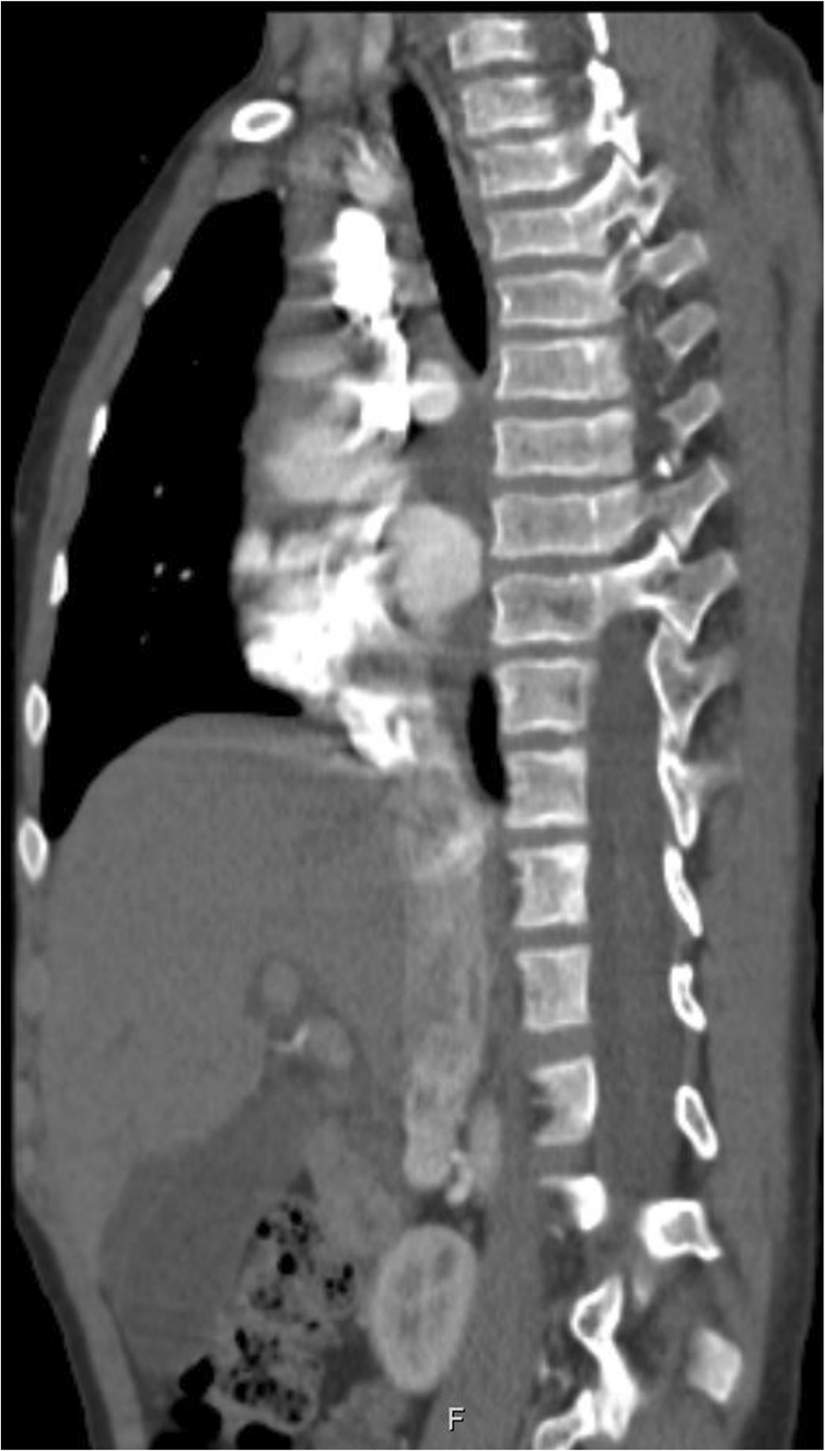
Lateral chest CT at 9 years showing platyspondyly

**Fig. 4:**
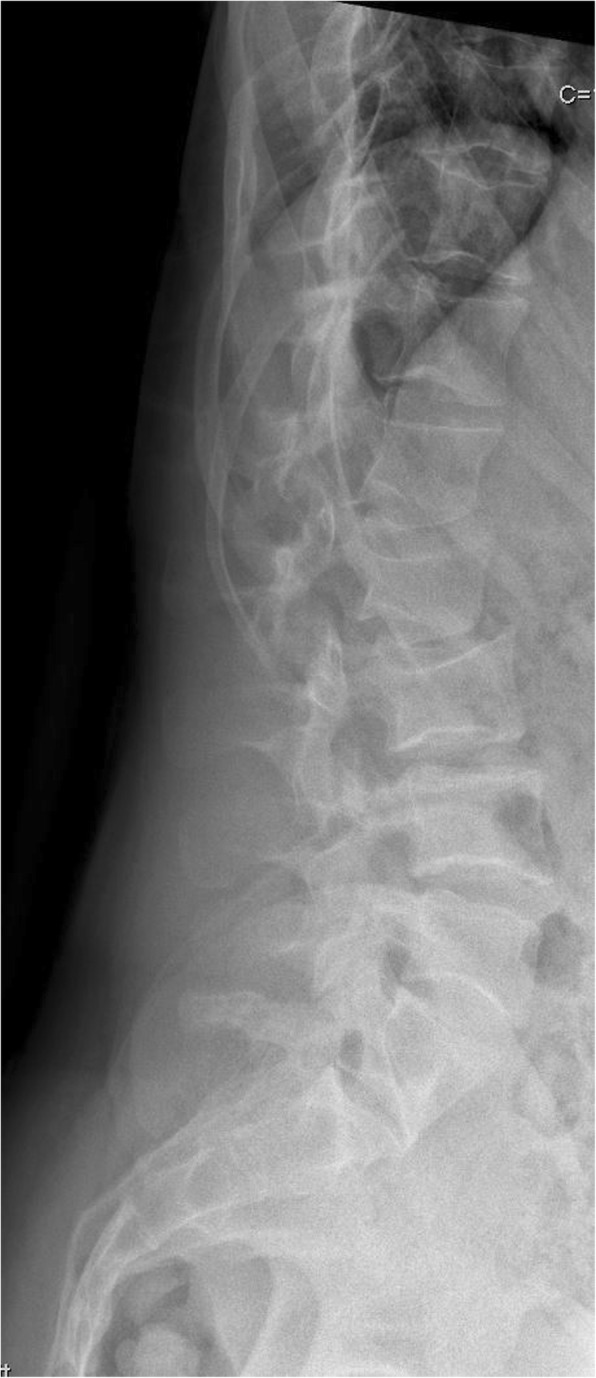
Lumbar spine at 20 years showing slight platyspondyly with biconcave endplates

He had a radius fracture when he was 15 years old. DEXA scan revealed osteopenia with an age-matched score of − 2. At age 20, he was 167 cm tall (2 cm below the 3rd percentile for his age and gender), and a whole body skeletal survey revealed scaphocephaly (Fig. 2), generally slender long bones and slender ribs. He had slight platyspondyly with biconcave endplates. There was slight scalloping of the posterior vertebral wall of some of the lumbar vertebras [11]. Reminescents of coronal clefts could not be excluded [12]. There were small olistheses in the coronal and sagittal planes, especially in the lumbar region, and wire fixation in the cervical spine. He had a flattened thoracic kyphosis (Figs. 2, 3 and 4). No significant scoliosis was noted. There were no signs of extra ossification centers in the calcaneus. CT of the aorta at age 19 also demonstrating the spine and hips showed dural ectasia and slight protrusion of acetabuli bilaterally [13].

The widespread arterial affection in addition to other clinical and radiological observations gave rise to the suspicion of another genetic connective tissue disorder rather than Larsen syndrome. One of the physicians at the resource center suspected that his diagnosis could be LDS based on his medical history. However, his hearing loss and extensive skeletal affection caused a somewhat unusual and more pronounced phenotype than what is typically seen in LDS, at least in the experience of this physician. He was then referred for a second opinion by a clinical geneticist, who confirmed the findings of arachnodactyly, mild short stature, pectus carinatum, bifid uvula, micrognathia, hypertelorism, down-slanting palpebral fissures, and wide, atrophic scars. High throughput sequencing (HTS) analysis of 34 genes associated with hereditary connective tissue disorders identified two sequence variants: a novel unclassified missense mutation, c.1361 T > C (p.Leu454Pro) in the gene for LDS type 2, *TGFBR2* (NM_003242.5), as well as a likely pathogenic nonsense mutation, c.115C > T (p.Gln39*) in *COL2A1* (NM_001844.4), a gene that encodes collagen type 2. Parental testing indicated that the variant in *TGFBR2* was de novo, while the variant in *COL2A1* was inherited from the father. No pathogenic or unclassified variant was identified in the gene for autosomal dominant Larsen syndrome, *FLNB*.

An ophthalmological reexamination revealed no new findings. The lens and the vitreous were clear, and there was no sign of retinal degeneration or other pathology in the posterior segment of the eyes. The axial length was in the normal range, indicating an eye globe of normal size.

## Discussion and conclusions

**Fig. 5:**
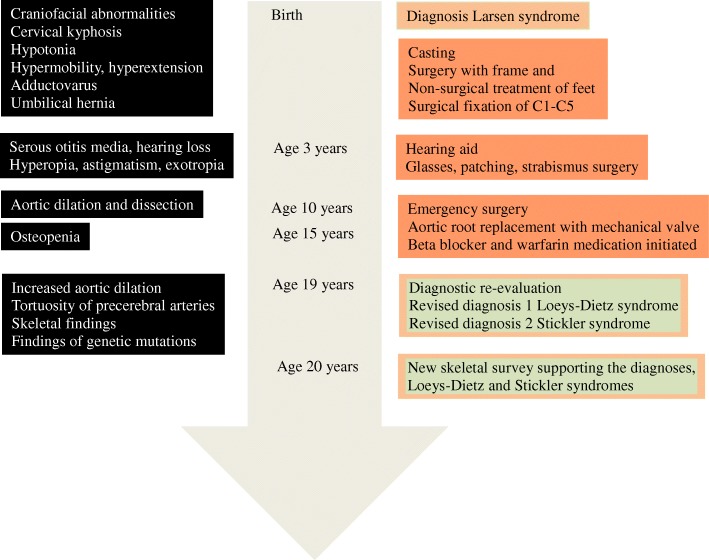
Timeline for the case report on clinical diagnosis of Larsen syndrome, Stickler syndrome and Loeys-Dietz syndrome in a 19 year old male

A timeline of the patient’s clinical history is indicated in Fig. 5. The suspicion that the patient could have another disorder than Larsen syndrome was supported by the presence of mutations in *TGFBR2* and *COL2A1* and by the absence of a mutation in the gene *FLNB*.

An autosomal recessive phenotype, formerly called autosomal recessive Larsen syndrome, has been reported with congenital heart defects, multiple joint dislocations, short stature, and craniofacial dysmorphism and is associated with mutations in the gene *B3GAT3,* which encodes a transmembrane protein with glucuronyltransferase properties [14, 15]. Findings in consanguineous families with *B3GAT3*-associated Larsen syndrome included heart failure due to congenital pulmonary stenosis and mitral insufficiency secondary to endocardial fibroelastosis. Congenital heart defects including bicuspid aortic valve with dilatation of the aortic root, patent foramen ovale, and ventricular septum defect as well as mild developmental delay were also reported [16, 17]. In the present case, the parents were not related. Sequencing of *B3GAT3* was not included in the available HTS gene panel.

According to the descriptions of LDS and MacCarricks classification, this patient’s diagnosis is LDS2, consistent with a de novo variant in *TGFBR2* as well as most of his clinical features. [5–9].

Intellectual disability in LDS is mostly associated with hydrocephalus or craniosynostosis [18]. Our patient had ventricular dilatation, scaphocephaly, and learning and mild behavioral problems, but no mental retardation. Further, the patient’s asthma and atopic eczema may also be part of LDS, which is associated with a high prevalence of asthma, allergies, or eczema [19].

The missense mutation that was identified in *TGFBR2* changes a highly conserved leucine residue located in the F-helix in the kinase domain of TGFBR2 [20]. The F-helix plays an important role in the function of TGFBR2, and a substitution of leucin at position 454 with proline might introduce a bend in the helix which would be devastating for the stability of the kinase domain. The fact that the patient’s mutation in *TGFBR2* was de novo supported the pathological predictions. *TGFBR2* mutations have also previously been reported in children with a clinical diagnosis of Larsen syndrome [21]. The major difference between autosomal dominant Larsen syndrome and LDS remains the occurrence of vascular abnormalities. While arterial involvement was reported in the previously defined, autosomal recessive form of Larsen syndrome, Bicknell et al. reported no arterial involvement in their description of 20, molecularly confirmed, *FLNB*-related cases [2]. It cannot be ruled out that some previous reported patients with a clinical diagnosis of Larsen syndrome and arterial involvement could have been misdiagnosed and actually have LDS, a diagnosis that was not yet described at the time [22].

The finding of a mutation in *COL2A1* was unexpected. Abnormalities in type II collagen result in a spectrum of autosomal dominant conditions, from prenatally lethal to relatively mild entities that may only become apparent in adulthood [12, 23]. 15 distinct phenotypes are listed in OMIM (Table 1), whereof the Stickler phenotype fits best with our patient. The genetically heterogeneous Stickler syndrome typically includes the ocular findings of myopia, cataract, and retinal detachment, in addition to conductive or sensorineural hearing loss and midfacial underdevelopment, cleft palate (isolated or as part of the Robin sequence), mild spondyloepiphyseal dysplasia, and/or precocious arthritis.

**Table 1:** Phenotypes related to mutations in *COL2A1* (OMIM +120140) [28]

Phenotype	Phenotype number	Inheritance
Achondrogenesis, type II or hypochondrogenesis	200610	AD
Avascular necrosis of the femoral head	608805	AD
Czech dysplasia	609162	AD
Epiphyseal dysplasia, multiple, with myopia and deafness	132450	AD
Kniest dysplasia	156550	AD
Legg-Calve-Perthes disease	150600	AD
Osteoarthritis with mild chondrodysplasia	604864	AD
Platyspondylic skeletal dysplasia, Torrance type	151210	AD
SED congenita	183900	AD
SEMD Strudwick type	184250	AD
Spondyloepiphyseal dysplasia, Stanescu type	616583	AD
Spondyloperipheral dysplasia	271700	AD
Stickler syndrome, type I, nonsyndromic ocular	609508	AD
Stickler syndrome, type I	108300	AD
Vitreoretinopathy with phalangeal epiphyseal dysplasia		

The patient fulfills the proposed diagnostic criteria for Stickler syndrome with bifid uvula, sensorineural hearing loss and a mutation known to be associated with Stickler syndrome [24]. He also has malar hypoplasia, retrognathia, and biconcave vertebral endplates. Bifid uvula, malar hypoplasia and retrognathia are also known features of LDS, but sensorineural hearing loss is not a known manifestation of this condition. While most of his radiographic findings are consistent with LDS [13], biconcave vertebral endplates have not, to our knowledge, been described in LDS. However, the affection of vertebral endplates and coronal clefts in infancy is a feature of Stickler syndrome [11].

The introduction of a premature stop codon in *COL2A1*, such as was found in this patient, is the most prevalent cause of *COL2A1*-associated Stickler syndrome. The nonsense mutation is located in exon 2, which is included in *COL2A1* isoform 1a, which is predominantly expressed in the vitreous of the eye in adult tissue [25]. In one family, mutations in exon 2 were reported to cause Stickler syndrome with minimal or no extraocular manifestations [26]. However, Tompson et al. recently reported a four generation pedigree with the exact same variant as in this patient [24] where the nonsense mutation displayed incomplete penetrance and/or variable age of onset with extraocular manifestations. These findings are in line with the findings for this patient and his father. Due to the patient’s young age, he may still develop ocular manifestations. Further, the presence of subtle, undiagnosed extraocular manifestations without subjective complaints in his father cannot be excluded. Incomplete penetrance and variable expressivity has also previously been reported in Stickler [12, 23, 27]. A further examination of the patient’s asymptomatic father and his parents could possibly have aided in further clarifying the role of the variant in *COL2A1,* but was not carried out.

The patient had most probably been misdiagnosed with Larsen syndrome 19 years earlier when LDS had not yet been described. His diagnosis was revised to LDS. We suggest that arterial involvement in a patient with a clinical diagnosis of Larsen syndrome, especially without a *FLNB* or *B3GAT3* mutation, should raise suspicions of misdiagnosis and prompt genetic testing for LDS. Clinicians should be aware of this, notably for the sake of correct genetic counseling and potentially life-saving vascular follow-up for patients and relatives.

He fulfills the proposed clinical criteria for Stickler syndrome, and the sequence variant in *COL2A1* may have an additional impact on this patient’s condition. To our knowledge, hearing loss and biconcave vertebral endplates have been reported in Stickler syndrome but not in LDS. We conclude that comorbidity with Stickler syndrome may have occurred in this patient, constituting a rare case of two concurrent hereditary connective tissue disorders. This case demonstrates that high throughput sequencing analysis using a targeted multi-gene panel is useful for differential diagnosis of genetic connective tissues disorders, as the spectra of clinical manifestations in several such disorders are highly overlapping. In complex cases with multiple clinical features not easily fitting into one single syndrome, overlapping may be due to multiple gene mutations, and direct proceeding to whole exome sequencing should be considered.

## Abbreviations

DEXA: Dual energy x-ray absorptiometry
ENT: Ear-nose-throat
HTS: High throughput sequencing analysis
LDS: Loeys-Dietz syndrome
TGF-β: Transforming growth factor beta
